# Assessing the epidemiology and seasonality of influenza among children under two hospitalized in Amman, Jordan, 2010‐2013

**DOI:** 10.1111/irv.12813

**Published:** 2020-11-11

**Authors:** Stephanie L. Rolsma, Danielle A. Rankin, Zaid Haddadin, Lubna Hamdan, Herdi K. Rahman, Samir Faouri, Asem Shehabi, John V. Williams, Najwa Khuri‐Bulos, Natasha B. Halasa

**Affiliations:** ^1^ Departments of Pediatrics Vanderbilt University Medical Center Nashville TN USA; ^2^ Vanderbilt University School of Medicine Nashville TN USA; ^3^ Department of Pediatrics Al Bashir Hospital Amman Jordan; ^4^ Department Pathology and Microbiology and Forensic Medicine Jordan University Amman Jordan; ^5^ Department of Pediatrics University of Pittsburgh School of Medicine Children’s Hospital of Pittsburgh of University of Pittsburgh Medical Center Pittsburgh PA USA; ^6^ Department of Pediatrics Jordan University Amman Jordan; ^7^ Institute for Global Health Vanderbilt University School of Medicine Nashville TN USA

**Keywords:** acute respiratory infections in MENA children, influenza in Jordanian children, influenza in the Middle East, influenza MENA region, influenza seasonality

## Abstract

**Background:**

The disease burden of influenza‐associated hospitalizations among children in Jordan is not well established. We aimed to characterize hospitalizations attributed to influenza in a pediatric population.

**Methods:**

We conducted a cross‐sectional study from our viral surveillance cohort in children under 2 years hospitalized with acute respiratory symptoms and/or fever from March 2010 to March 2013. We collected demographic and clinical characteristics, and calculated the frequency of children who met the severe acute respiratory illness (SARI) criteria. Nasal specimens were tested using real‐time reverse transcriptase polymerase chain reaction to detect influenza A, B, or C. Further subtyping for influenza A‐positive isolates was conducted.

**Results:**

Of the 3168 children enrolled in our study, 119 (4%) were influenza‐positive. Influenza types and subtypes varied by season but were predominantly detected between December and February. Codetection of multiple respiratory pathogens was identified in 58% of children with the majority occurring among those <6 months. Bronchopneumonia and rule‐out sepsis were the most common admission diagnoses, with influenza A accounting for over 2/3 of children with a rule‐out sepsis admission status. One‐third of children under 6 months compared to 3/4 of children 6‐23 months met the SARI criteria.

**Conclusions:**

Influenza was an important cause of acute respiratory illness in children under 2 years. Children <6 months had the highest burden of influenza‐associated hospitalizations and were less likely to meet the SARI global surveillance case definition. Additional surveillance is needed in the Middle East to determine the true influenza burden on a global scale.

## 1 BACKGROUND

Acute respiratory infections (ARI) are the leading cause of morbidity and mortality of children under 5 years outside the neonatal period.^1^ The majority of ARIs are viral in origin, with influenza substantially contributing to outpatient and emergency department visits, hospitalizations, and even deaths in these children.^1^ Influenza is a vaccine‐preventable disease and impacts all age‐groups, with the highest risk of influenza‐related complications in children under 2 years, adults older than 65 years, pregnant women, and individuals with underlying medical conditions.^2^ Worldwide, although influenza hospitalizations among the pediatric population are known to inflict a large burden, the exact number of global cases and hospitalizations attributed to influenza is not well described.^1^, ^3^

In 2008, a systematic review estimated the global incidence of influenza in children under 5 years to be 90 million cases, with approximately one million severe cases.^1^ The uncertainties of global pediatric influenza‐associated hospitalization burden estimates may be posited to the lack of standard worldwide reporting and testing.^4^ In response to the 2009 H1N1 pandemic and in an attempt to overcome the influenza surveillance gap, the World Health Organization (WHO) launched an initiative in 2011 to develop global standards for influenza surveillance, including a global case definition of severe influenza.^5^ The case definition was intended to capture hospitalizations related to influenza and is known as severe acute respiratory infection (SARI), defined as an acute respiratory illness with a measured temperature of ≥38° Celsius and cough, with illness onset within the past 10 days, and hospitalization.^5^ Although a standardized approach for influenza surveillance has been developed, the epidemiology in many parts of the world, including the Middle East North Africa (MENA) region, remains unclear, especially among young children.^1^, ^3^, ^4^, ^6^, ^7^

Since 2007, Jordan has participated in sentinel‐site surveillance with the Eastern Mediterranean Acute Respiratory Infection Surveillance (EMARIS) network.^8^ One study conducted in Jordan identified that 9% of all patients who met the SARI case definition were influenza‐positive, with 3% of influenza‐associated deaths, of which all occurred among pediatric patients.^4^ Additional influenza research has been conducted in Jordan, but many of these studies have only included individuals who met the SARI case definition and have not solely focused on a pediatric population.^4^ Therefore, our study aimed to evaluate and describe the epidemiology, seasonality, and clinical characteristics of influenza‐associated hospitalizations in Jordanian children under 2 years, who presented to a large government hospital with fever and/or respiratory symptoms over three full respiratory seasons.

## 2 METHODS

### 2.1 Study design

From March 13, 2010, to March 31, 2013, we conducted a prospective year‐round viral surveillance study of children <2 years who were hospitalized with acute respiratory symptoms and/or fever within 48‐hours of hospitalization at Al‐Bashir Hospital in Amman, Jordan.^9^ Enrollment occurred Sunday through Thursday and children with chemo‐associated neutropenia and/or newborns never discharged from the hospital were excluded from the study (detailed inclusion/exclusion criteria are previously published).^9^ Written informed consent was obtained from parents or legal guardians prior to enrollment into our study.^9^ The study was approved by the Institutional Review Boards at the University of Jordan, the Jordan Ministry of Health, and Vanderbilt University.^9^

### 2.2 Study site

During the study period, Al‐Bashir Hospital had a total of 185 pediatric beds (120 pediatric and 65 neonatal intensive care unit) and 11 230 hospitalizations among children <2 years. ^7^, ^9^, ^10^, ^11^ Al‐Bashir is one of three major government run hospitals that services the population of Amman (capital and largest city in Jordan [>2 million persons]), with over 60% of the pediatric care occurring at this hospital.^7^, ^9^, ^10^, ^11^ As part of the government policy, all Jordanian children under 6 years are provided no‐cost medical care, regardless of insurance status, at Al‐Bashir Hospital.^7^, ^9^, ^10^, ^11^

### 2.3 Data and specimen collection

After obtaining informed consent, trained research personnel collected nasal and throat swabs from all enrolled children.^7^, ^9^, ^10^, ^11^ Parents/guardians were interviewed to obtain the child's demographic characteristics and medical and social histories using a standardized questionnaire.^7^, ^9^, ^10^, ^11^ All interviews were conducted in Arabic using a standardized case report form and transcribed into English.^7^, ^9^, ^10^, ^11^ After children were discharged, medical records were abstracted for the following: oxygen use, intensive care unit (ICU) stay, mechanical ventilation, length of stay in the hospital, and discharge status.^10^ Complete details on the methods of data collection are explained in a previous publication.^10^

We inputted and stored all data in a secure REDCap™ (Research Electronic Data Capture, Vanderbilt University, Nashville, TN, USA) database.^9^, ^12^ Data quality checks were performed on a minimum of 10% of the charts, and data from all case report forms were verified after entry.^7^, ^9^

### 2.4 Laboratory methods

Nasal and throat swabs were combined into transport medium (M4RT^®^, Remel), aliquoted into MagMAX™ Lysis/Binding Solution Concentrate (Life Technologies), snap‐frozen, stored at −80°C, and shipped on dry ice to Nashville, TN, USA.^7^, ^9^, ^10^, ^11^ Testing of original and lysis buffer aliquots was conducted through real‐time reverse transcriptase polymerase chain reaction (RT‐PCR) for eleven respiratory viruses: influenza A, B, and C; parainfluenza virus (PIV) 1, 2, and 3; human metapneumovirus (HMPV); respiratory syncytial virus (RSV); human rhinovirus (HRV); adenovirus; and Middle East respiratory syndrome coronavirus (MERS‐CoV).^7^, ^9^, ^10^, ^11^ Influenza A was further subtyped as H1N1 or H3N2.

### 2.5 SARI criteria/case definitions

We categorized children into two groups: (a) children who met SARI criteria and (b) children that did not meet SARI criteria. Qualifying characteristics were extracted from the interview‐derived questionnaires and medical chart abstractions. The fever component of SARI was met if the child had one of the following: self‐reported history of fever during current illness, temperature of ≥38° Celsius recorded at admission, and/or an admission or discharge diagnosis of fever.^10^ Children were recorded to meet the cough component if it was self‐reported as a symptom and/or was recorded as an admission or discharge diagnosis. Illness duration was captured on the standardized questionnaire, and the duration component of SARI was met if the child had illness duration of 10 days or less at enrollment.^10^

Intensive care unit stay included children who were transferred to the ICU during the admission or were admitted directly. Children were categorized as rule‐out sepsis (ROS) if they had the admission diagnosis of “rule‐out sepsis” or “febrile neonate”.^7^

### 2.6 Data analysis

Descriptive statistics are reported as frequency or median and interquartile range where appropriate. We used Pearson chi‐square and Fisher's exact tests to compare categorical variables and two‐sample *t* tests allowing for unequal variances for continuous variables. Seasonality and trends of influenza type and subtype are evaluated using an epidemiologic curve by the date of specimen collection. All analyses were conducted using statistical software StataIC 16.0 (StatCorp LLC).

## 3 RESULTS

### 3.1 Study population and demographics

From March 2010 to March 2013, we identified 3793 children eligible for enrollment; 618 (16%) children had parent/guardian refuse to study participation, three were deemed ineligible after enrollment due to being older than 2 years, and four children had a diagnosis of meningitis.^7^ Our final cohort included 3168 children, of which 119 (4%) were influenza‐positive.

### 3.2 Demographics and clinical characteristics

The most common symptoms reported were cough, fever, wheezing, and shortness of breath (SOB); children primarily had an admission diagnosis of either bronchopneumonia or ROS (Table 1). Compared to influenza‐negative children, influenza‐positive children were older and more likely to present with fever. Overall, influenza‐positive children were less likely to be administered oxygen, but had a higher proportion of death, but these were not statistically significant (Table 1). Of those children who died, two had influenza A H3N2 and one had influenza A H1N1pdm09. Interestingly, two of the children had a serious comorbid condition, osteogenesis imperfecta. Of note, only six children were reported to have influenza vaccination and none were influenza‐positive.

**Table 1:** Demographic and clinical characteristics of Jordanian children hospitalized with ARI over three respiratory seasons

Characteristics	Influenza Positive (n = 119)	Influenza Negative^a^ (n = 3049)	*P*‐value	Influenza, Only (n = 50)	Influenza Codetection (n = 69)	*P*‐value
Age, months (median [IQR])	5.4 [1.8‐12.5]	3.5 [1.6‐8.4]	**.003**	6.6 (1.6‐15.2)	5.5 (1.9‐11.9)	.308
Sex, male	71 (60.0)	1841 (60.4)	.875	24 (48.0)	47 (68.1)	**.027**
Cesarean section	36 (30.3)	857 (28.1)	.610	12 (24.0)	24 (34.8)	.206
Premature, <37 wks	16 (13.5)	434 (14.2)	.809	5 (10.0)	11 (15.9)	.348
Birthweight, kg (median [IQR])	3 (2.7‐3.5)	3 (2.5‐3.5)	.617	3.0 (2.5‐3.5)	3.0 (2.7‐3.5)	.485
Underlying medical condition	20 (16.8)	355 (11.6)	.087	9 (18.0)	11 (15.9)	.767
Breastfeeding Hx.	96 (80.7)	2565 (84.1)	.313	40 (80.0)	56 (81.2)	.874
No. days reported sick (median [IQR])	3.0 (2.0‐7.0)	3.0 (2.0‐4.0)	.965	3.0 (2.0‐7.0)	3.0 (2.0‐5.0)	.101
No. of household members (median [IQR])	6.0 (4.0‐7.0)	5.0 (4.0‐7.0)	.316	6.0 (4.0‐7.0)	6.0 (5.0‐7.0)	.538
Smoke exposure, nargila, or cigarette	91 (76.5)	2334 (76.6)	.984	40 (80.0)	51 (73.9)	.440
Influenza vaccine	0 (0.0)	6 (0.2)	.369^b^	0 (0.0)	0 (0.0)	‐
Met SARI criteria	63 (52.9)	1198^c^ (39.3)	**.003**	28 (56.0)	35 (50.7)	.569
Symptoms						
Fever	86 (72.3)	1676 (55.0)	**<.001**	42 (84.0)	44 (63.8)	**.015**
Cough	90 (75.6)	2276 (74.7)	.809	34 (68.0)	56 (81.2)	.099
Congestion	0 (0.0)	26 (0.9)	.623^b^	0 (0.0)	0 (0.0)	‐
Runny nose	2 (1.7)	51 (1.7)	1.000^b^	2 (4.0)	0 (0.0)	.174^b^
Vomiting	12 (10.1)	510 (16.7)	.055	5 (10.0)	7 (10.1)	.979^b^
Diarrhea	14 (11.8)	303 (9.9)	.515	7 (14.0)	7 (10)	.519
Apnea	0 (0.0)	10 (0.3)	.531^b^	0 (0.0)	0 (0.0)	‐
Shortness of breath	63 (52.9)	1769 (58.0)	.271	18 (36.0)	45 (65.2)	**.002**
Wheezing	65 (54.6)	1692 (55.5)	.851	24 (48.0)	41 (59.4)	.217
Seizures/ab. movement	4 (3.4)	121 (4.0)	1.000^b^	1 (2.0)	3 (4.4)	.638^b^
Admitting diagnosis						
Bronchiolitis	14 (11.8)	533 (17.5)	.106	0 (0.0)	14 (20.3)	**<.001** ^b^
Bronchopneumonia	48 (40.3)	972 (31.9)	.053	23 (46.0)	25 (36.2)	.284
Pneumonia	13 (10.9)	381 (12.5)	.610	3 (6.00)	10 (14.5)	.233^b^
Febrile seizure	3 (2.5)	80 (2.6)	1.000^b^	1 (2.0)	2 (2.9)	1.000^b^
Rule‐out sepsis	31 (26.1)	881 (28.9)	.501	16 (32.0)	15 (21.7)	.208
Severity						
Length of stay, days (median [IQR])	5.0 (3.0‐7.0)	5.0 (3.0‐6.5)	.155	5.0 (3.0‐6.0)	5.0 (3.0‐7.0)	.895
Admission to ICU	10 (8.4)	274 (9.0)	.827	3 (6.0)	7 (10.1)	.517^b^
Admin. oxygen	30 (25.2)	983^d^ (32.2)	.092	9 (18.0)	21 (30.4)	.123
Mechanical ventilation	1 (0.8)	110^e^ (3.7)	.128^b^	1 (2.0)	0 (0.0)	.420^b^
Died	3 (2.5)	28^e^ (0.4)	.111^b^	1 (2.0)	2 (2.90)	1.000^b^

Note

Data are n (%), unless otherwise specified. *P*‐values were calculated using Pearson chi‐square test for categorical variables and independent samples *t* test for continuous variables.

Abbreviations: Ab., abnormal; Admin., administration; ARI, acute respiratory infection; Hx., history; Kg, kilogram; No., number; SARI, severe acute respiratory infection (ie, defined as fever, cough, and symptom onset in past 10 d).

Bold values are statistically significant <.05.

^a^ Includes children who were virus negative.

^b^ Fisher's exact test.

^c^ n = 3045.

^d^ n = 3018.

^e^ n = 3017.

### 3.3 Codetection with other viruses

Among influenza‐positive children, 58% had additional co‐pathogens detected. Children with influenza codetection with other respiratory viruses were more likely to be male compared to children with influenza detection alone (Table 1). Whereas children with only influenza detection were more likely to present with fever, they were less likely to have SOB. Interestingly, no children with influenza detection alone had an admitting diagnosis of bronchiolitis (Table 1).

### 3.4 Influenza types and seasonality

**FIGURE 1:**
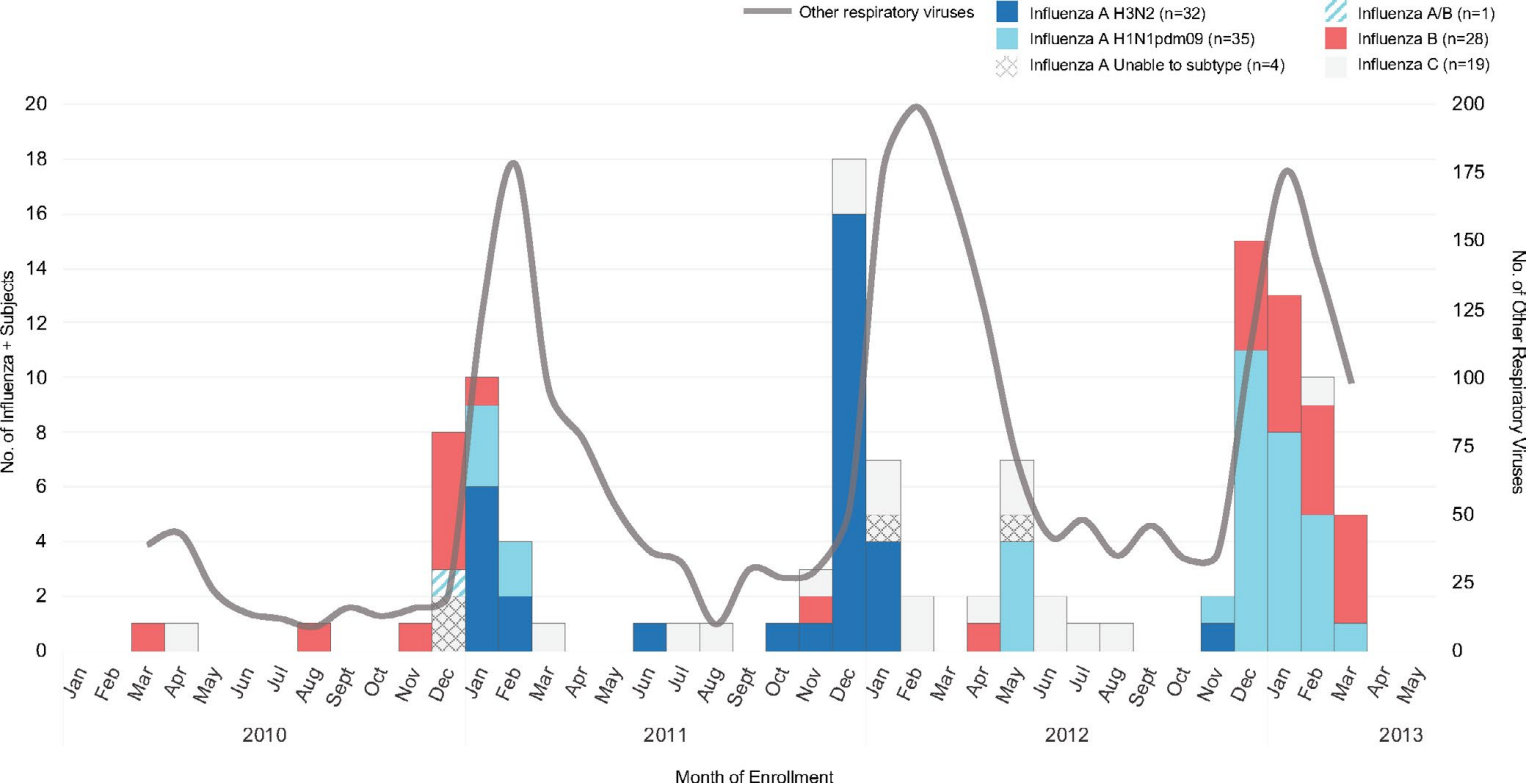
Seasonality of Influenza‐Positive Children compared to Other Respiratory Viruses Detected from March 2010 to March 2013, stratified by type/subtype

Throughout our study, influenza types and subtypes varied by season; however, the majority of influenza‐positive cases were detected in the months of December through February (Figure 1). The majority of the cases were influenza A (H1N1pdm09 =35; H3N2 =32; unable to subtype  =4), followed by influenza B (n  =28), influenza C (n  =19), and influenza A/B (n  =1). From December 2011 to January 2012, influenza A H3N2 was the most frequently identified subtype among children. During the following influenza season (December 2012‐March 2013), influenza A H1N1pdm09 was the predominant subtype detected, followed by influenza B. In February 2011 and 2012, other respiratory viruses peaked as influenza cases declined (Figure 1).

### 3.5 Clinical presentation, codetection, and admission diagnosis stratified by age

We stratified influenza‐positive children into three age‐groups: under 6 months; 6‐11 months; and 12‐23 months. Compared to the 6‐11 months age‐group, children under 6 months with influenza were more likely to have a history of breastfeeding, but less likely to be premature and had higher birthweight (Table 2). In addition, they were less likely to present with SOB and wheezing and had longer hospital stay. Compared to the 12‐23 months of age‐group, children under 6 months with influenza were more likely to be male and less likely to have an underlying medical condition and present with vomiting. When compared to both other age‐groups, the under 6 months children were less likely to have fever, cough, and meet the SARI case definition (Table 2). In addition, all three mortalities were in children under 6 months.

**Table 2:** Demographic and clinical characteristics of influenza‐positive Jordanian children hospitalized over three respiratory seasons, stratified by age‐group

Characteristics	Age‐Group				
	<6 mo^a^ (n = 62)	6‐11 mo (n = 24)	*P‐*value	12‐23 mo (n = 33)	*P*‐value
Sex, male	42 (67.7)	17 (70.8)	.782	12 (36.4)	**.003**
Cesarean section	20 (32.3)	7 (29.2)	.782	9 (27.3)	.615
Premature, <37 wks	5 (8.1)	6 (25.0)	**.035**	5 (15.2)	.284
Birthweight, kg (median [IQR]))	3.1 (2.7‐3.5)	2.8 (2.1‐3.3)	**.044**	3.2 (2.8‐3.5)	.676
Underlying medical condition	7 (11.3)	3 (12.5)	1.000^b^	10 (30.3)	**.021**
Breastfeeding Hx.	54 (87.1)	16 (66.7)	**.029**	26 (78.8)	.290
No. days reported sick (median [IQR])	2.0 (1.0‐5.0)	3.0 (1.5‐6.0)	.651	4.0 (2.0‐7.0)	.151
No. of household members (median [IQR]))	5.0 (4.0‐7.0)	5.0 (4.5‐6.5)	.646	6.0 (5.0‐7.0)	.214
Smoke exposure, nargila or cigarette	51 (82.3)	17 (70.8)	.243	23 (69.7)	.160
Met SARI criteria	20 (32.3)	18 (75.0)	**<.001**	25 (75.8)	**<.001**
Influenza, codetection	38 (61.3)	14 (58.3)	.801	17 (51.5)	.358
Symptoms					
Fever	37 (59.7)	20 (83.3)	**.037**	29 (87.9)	**.004**
Cough	39 (62.9)	22 (91.7)	**.008**	29 (87.9)	**.010**
Fever & cough	17 (27.4)	18 (75.0)	**<.001**	25 (75.8)	**<.001**
Runny nose	1 (1.6)	0 (0)	1.000^b^	1 (3.0)	1.000^b^
Vomiting	2 (3.2)	3 (12.5)	.130^b^	7 (21.2)	**.008** ^b^
Diarrhea	8 (12.9)	1 (4.2)	.434^b^	5 (15.2)	.762
Shortness of breath	27 (43.6)	17 (70.8)	**.023**	19 (57.6)	.193
Wheezing	26 (41.9)	20 (83.3)	**.001**	19 (57.6)	.146
Seizures/abnormal movement	0 (0)	2 (8.3)	.076^b^	2 (6.1)	.118
Severity					
Length of stay, days (median [IQR])	5.0 (3.0‐8.0)	4.0 (2.5‐5.0)	**.002**	5.0 (3.0‐6.0)	.171
Admission to ICU	8 (12.9)	0 (0)	.099^b^	2 (6.1)	.486
Administered oxygen during hospitalization	18 (29.0)	6 (25.0)	.708	6 (18.2)	.247
Mechanical ventilation	1 (1.6)	0 (0)	1.000^b^	0 (0)	1.000^b^
Died	3 (4.8)	0 (0)	.557	0 (0)	.549

Note

Data are n (%), unless otherwise specified. *P*‐values were calculated using Pearson chi‐square test for categorical variables and independent samples *t* test for continuous variables.

Abbreviations: Ab., abnormal; Admin., administration; Kg, kilogram; No., number; SARI, severe acute respiratory infection (ie, defined as fever, cough, and symptom onset in past 10 d). 1

^a^ Denotes referent group for pairwise comparisons.

^b^ Fisher's exact test.

**FIGURE 2:**
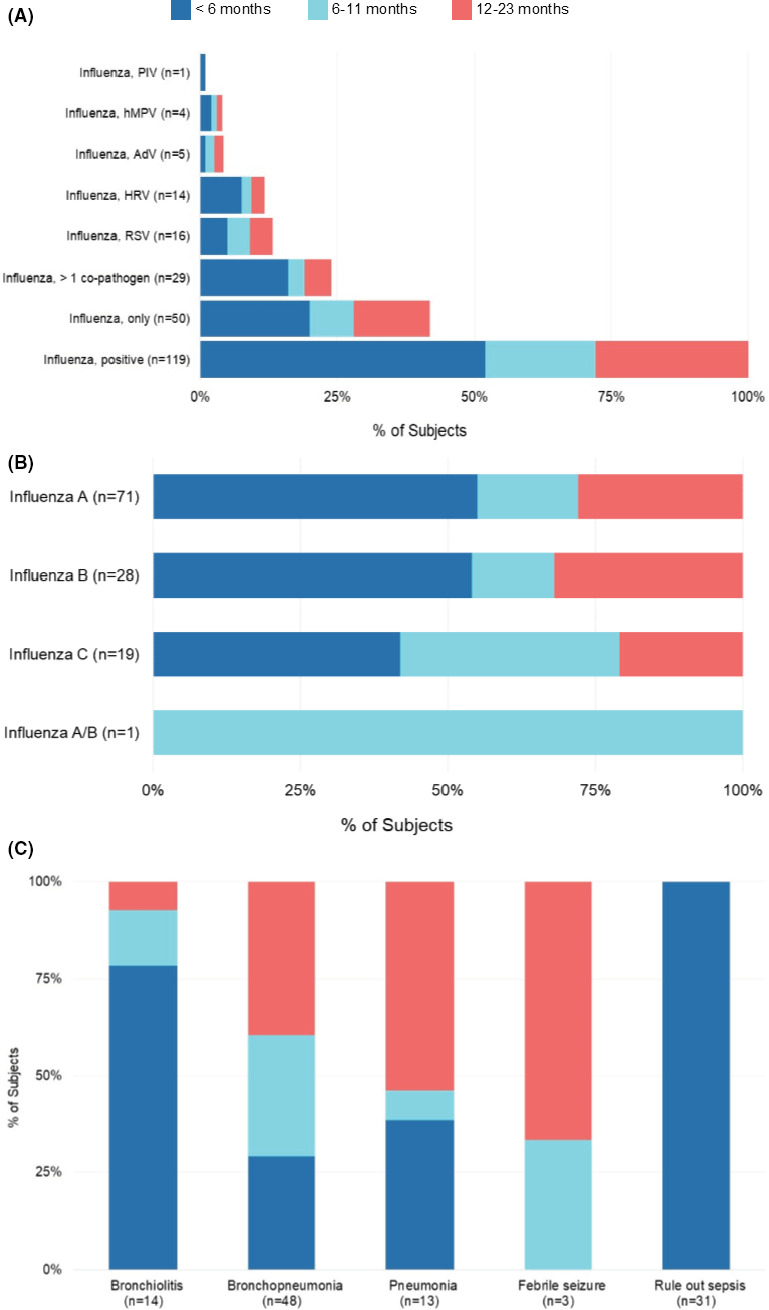
Proportion of Single and Codetection of Influenza (A), Type of Influenza (B), and Admitting Diagnosis (C) of Hospitalized Jordanian Children, by Age‐Group

**FIGURE 3:**
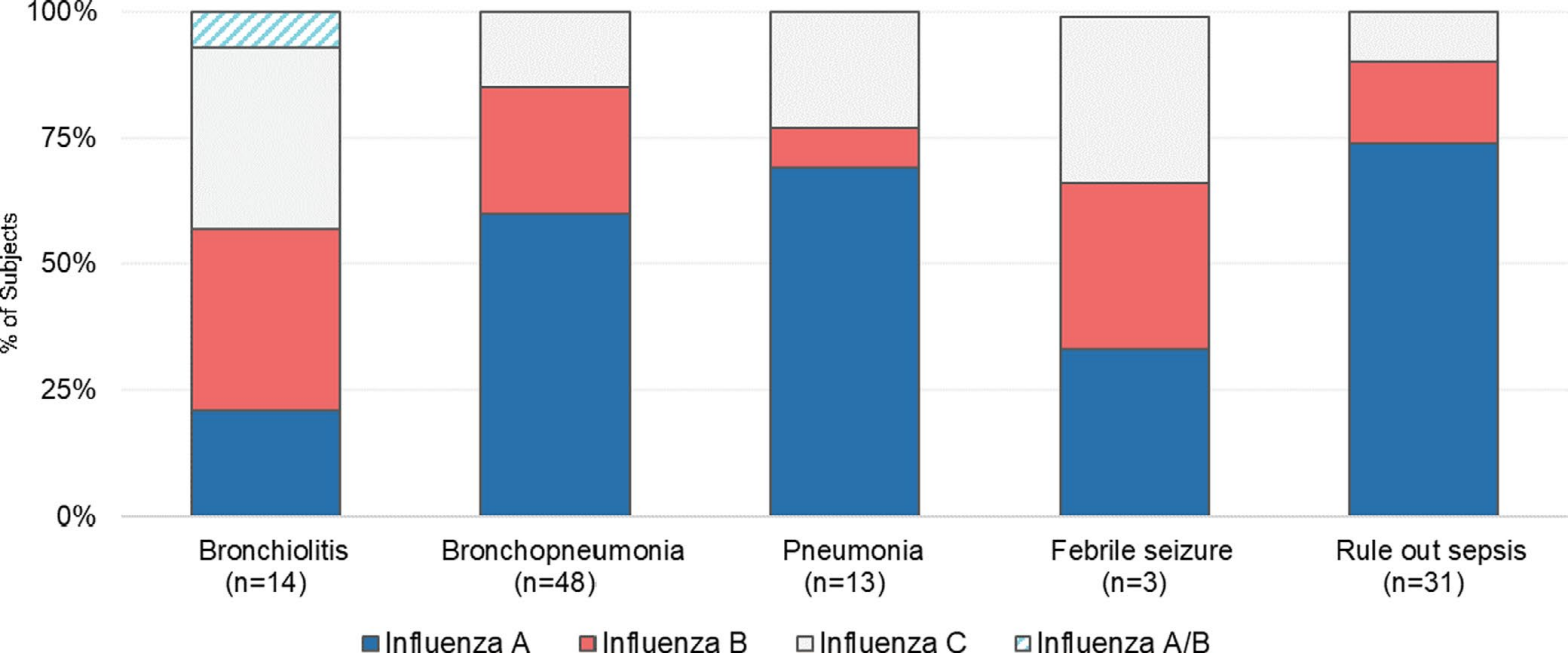
Admitting Diagnosis of Hospitalized Jordanian Children with Influenza, by Influenza Type

Of the influenza‐positive children with multiple respiratory pathogens detected, 24% had greater than one co‐pathogen, with the majority of codetection occurring among children under 6 months (66%) (Figure 2A). Respiratory syncytial virus was the co‐pathogen detected in 14% of influenza‐positive children with no differences between age‐groups. Overall, influenza A and B were more commonly detected in children under 6 months, while influenza C was equally detected in children under 6 months and 6‐11 months (Figure 2B). Bronchiolitis and ROS were the most common admission diagnoses given to children under 6 months compared to children 6 months and older (Figure 2C), with influenza A accounting for >70% of all children with an ROS admission status (Figure 3). In comparison, influenza type was moderately similar among those children with a bronchiolitis admitting diagnosis.

## 4 DISCUSSION

Our 3‐year prospective viral surveillance study of 3168 Jordanian children identified influenza in 4% of all total acute respiratory hospitalizations, and even higher during winter months. One‐half of the influenza‐associated hospitalizations were among children younger than 6 months, and influenza A was the most common subtype identified. Our findings suggest that influenza is a common respiratory pathogen impacting young children throughout the winter season in Jordan, and these children are not routinely vaccinated against influenza, further placing an emphasis on the importance of surveillance and prevention measures, such as annual vaccination.

Influenza A/H1N1pdm09 and H3N2 were the most predominant subtypes throughout the study period, with influenza B primarily co‐circulating in the 2012‐2013 winter season. The subtypes we identified circulating over the 3 years are consistent with other population‐based surveillance studies in the MENA region and Northern Hemisphere.^4^, ^13^, ^14^, ^15^ In a 6‐year sentinel surveillance study in Jordan of all ages, influenza A H3N2 predominately circulated in 2011‐2012 and H1N1pdm09 was more common in 2012‐2013.^4^ Similarly, in a systematic review from 2010‐2016, 61.9% of cases in the MENA region were attributed to influenza A H1N1pdm09, with a primary peak from Jordan regularly occurring in January.^14^ However, these studies evaluated cases who met the SARI criteria and categorized pediatric cases into age‐groups that do not provide sufficient epidemiological information on children under 2 years. Therefore, pediatric studies focusing on influenza burden are important for education campaigns and uptake of annual influenza vaccination.^4^ This information can help provide information to inform MENA initiatives targeted toward children.

In our study, fever was an important clinical criterion with influenza‐positive children, particularly in children 6‐23 months. Specifically, fever seems to be a unique component of the presentation to children who only had influenza detected. While cough was another common presenting symptom, it was much less common in children under 6 months was significantly more commonly detected in children with codetection. Moreover, the combination of fever and cough together was rarely seen in these young children. Therefore, our results imply that fever may be a useful clinical metric to determine which pediatric patients should be tested for influenza, especially during winter months when influenza circulation is more likely. Cough may be a less useful indicator, and when it is a required criterion to test for influenza, it may actually lead to an underestimation, particularly in young infants. In a comparable study from the United States in children that enrolled 160 children under 5 years with influenza who were hospitalized for fever or acute respiratory symptoms, fever and cough were common presenting symptoms.^3^ However, both cough and fever plus cough were also less common in children younger than 6 months. Rhinorrhea was much more common in this study (83% compared to 1.7% in our study) but this could be attributed to the wider age range enrolled in this study. In another similar study from China in 480 children with influenza who were <15 years old and hospitalized for ARI, fever was a common presenting symptom in children under 24 months but was less common in younger infants.^16^ Therefore, the inclusion of fever or cough for viral surveillance is important but both should not be required for surveillance studies that are trying to estimate the exact influenza burden to avoid underestimation of influenza in young children.

Several prior epidemiological studies in this region have focused on children with SARI, which may also result in a misestimation of the true burden of influenza in the pediatric population given the specific case definition that requires both fever and cough. This is supported by results from our study where approximately half of influenza cases met SARI case definitions, and only one‐fifth of influenza cases in the <6‐month group met SARI case definitions. In other reports in the region, influenza surveillance studies often use the SARI case definition, include adults as well as children, and do not identify clinical characteristics of infection in influenza‐positive children.^4^, ^8^, ^15^, ^17^, ^18^, ^19^ Therefore, caution should be used when using SARI for enrollment criteria and estimating the true burden of influenza disease.

A high index of suspicion must also be maintained for influenza infection as infants may present differently compared to older children and adults. In our study, many infants eventually diagnosed with influenza were admitted with a rule‐out sepsis diagnosis. A study of admission diagnoses in 401 children under 16 years who were hospitalized for influenza in Finland also showed that suspected sepsis was a common admission diagnosis in children <6 months.^20^ Similar to our study, the previously discussed US study reported bronchiolitis, pneumonia, and fever/suspected sepsis as some of the most common diagnoses; however, this study only recorded discharge rather than our study which recorded admission diagnoses.^3^ Importantly, children under 6 months who were influenza‐positive were the only children who died and had a longer length of stay than other age‐groups, highlighting the risks of severe disease in the younger pediatric population. Further studies may be needed to determine the importance of influenza in this population, especially considering that few studies have been conducted in this region and treatment for influenza virus infection is available and early diagnosis and treatment improves outcomes.

A major strength of our study was that we conducted a prospective 3‐year viral surveillance study in children <2 years in a major government hospital in Jordan. All specimens from enrolled children were tested via RT‐qPCR for viral detection, which is more sensitive than detection methods used in previous studies. Also, since research personnel enrolling children were different than the clinical providers hospitalizing the patients, differential surveillance bias was reduced. Although we have many strengths, we do have some notable limitations. First, our surveillance efforts were restricted to Al Bashir Hospital in Amman, Jordan. The hospital services families of low and middle income and is not the only government hospital in Jordan. Therefore, our results are not generalizable to all children under 2 years in Jordan. Additionally, our study allowed for re‐enrollment of children from season to season; therefore, there may be children who were captured in our surveillance more than once. Finally, our study enrolled children only 5 days per week, thus indicating our results may be an underestimation of the true influenza burden in Amman, Jordan.

In conclusion, the burden of influenza requiring hospitalization among children in Amman, Jordan, was highest in those younger than 6 months, with influenza A being the most predominant type circulating annually. Our results suggest that using SARI criteria may exclude cases of influenza illness that can nonetheless be quite severe in patients under 2 years of age. Modifying the SARI surveillance definition to include either fever or cough for young children who present with different clinical symptoms may impact the annual influenza rates, which in turn can assist with implementation of prevention strategies on a global scale. Maternal vaccination and targeting influenza vaccination in children older than 6 months may help reduce burden of influenza in the region.

## CONFLICT OF INTEREST

Natasha Halasa, MD, MPH receives grant support from Sanofi, Quidel, and speaker compensation from an education grant supported by Genentech. Sanofi also donated vaccines and influenza antibody testing for influenza vaccine trial. John Williams, MD is on the scientific board for Quidel, Independent Data Safely Monitoring Committee, GlaxoSmithKline, scientific advisory board ID Connect.

## AUTHOR CONTRIBUTIONS

**Stephanie L Rolsma:** Writing‐original draft (lead); Writing‐review & editing (lead). **Danielle A Rankin:** Formal analysis (lead); Writing‐original draft (lead); Writing‐review & editing (lead). **Zaid Haddadin:** Writing‐review & editing (equal). **Lubna Hamdan:** Writing‐review & editing (equal). **Herdi K Rahman:** Formal analysis (equal); Writing‐review & editing (equal). **Samir Faouri:** Conceptualization (equal); Methodology (equal); Supervision (equal); Writing‐review & editing (equal). **Asem Shehabi:** Investigation (equal); Methodology (equal); Supervision (equal); Writing‐review & editing (equal). **John Williams:** Methodology (equal); Writing‐review & editing (equal). **Najwa Khuri‐Bulos:** Methodology (equal); Supervision (equal); Writing‐review & editing (equal). **Natasha B Halasa:** Conceptualization (lead); Investigation (lead); Methodology (lead); Project administration (lead); Supervision (lead); Writing‐original draft (supporting); Writing‐review & editing (supporting).

## Footnotes

1 Bold values are statistically significant <.05.

## References

[1] Nair H , Brooks WA , Katz M , et al. Global burden of respiratory infections due to seasonal influenza in young children: a systematic review and meta‐analysis. Lancet. 2011;378(9807):1917‐1930. 22078723 10.1016/S0140-6736(11)61051-9

[2] Al Awaidy S , Althaqafi A , Dbaibo G , Middle East/North Africa Influenza Stakeholder N . A Snapshot of influenza surveillance, vaccine recommendations, and vaccine access, drivers, and barriers in selected middle eastern and North African Countries. Oman Med J. 2018;33(4):283‐290. 30038727 10.5001/omj.2018.54 PMC6047181

[3] Poehling KA , Edwards KM , Weinberg GA , et al. The underrecognized burden of influenza in young children. N Engl J Med. 2006;355(1):31‐40. 16822994 10.1056/NEJMoa054869

[4] Al‐Abdallat M , Dawson P , Haddadin AJ , et al. Influenza hospitalization epidemiology from a severe acute respiratory infection surveillance system in Jordan, January 2008‐February 2014. Influenza Other Respir Viruses. 2016;10(2):91‐97. 26505620 10.1111/irv.12354 PMC4746565

[5] Fitzner J , Qasmieh S , Mounts AW , et al. Revision of clinical case definitions: influenza‐like illness and severe acute respiratory infection. Bull World Health Organ. 2018;96(2):122‐128. 29403115 10.2471/BLT.17.194514 PMC5791775

[6] Elhakim M , Hafiz Rasooly M , Fahim M , et al. Epidemiology of severe cases of influenza and other acute respiratory infections in the Eastern Mediterranean Region, July 2016 to June 2018. J Infect Public Health. 2020;13(3):423‐429. 31281105 10.1016/j.jiph.2019.06.009 PMC7102678

[7] Khuri‐Bulos N , Lawrence L , Piya B , et al. Severe outcomes associated with respiratory viruses in newborns and infants: a prospective viral surveillance study in Jordan. BMJ Open. 2018;8(5):e021898. 10.1136/bmjopen-2018-021898 PMC5961648 29780032

[8] Horton KC , Dueger EL , Kandeel A , et al. Viral etiology, seasonality and severity of hospitalized patients with severe acute respiratory infections in the Eastern Mediterranean Region, 2007–2014. PLoS One. 2017;12(7):e0180954. 28704440 10.1371/journal.pone.0180954 PMC5509236

[9] Halasa N , Williams J , Faouri S , et al. Natural history and epidemiology of respiratory syncytial virus infection in the Middle East: Hospital surveillance for children under age two in Jordan. Vaccine. 2015;33(47):6479‐6487. 26314623 10.1016/j.vaccine.2015.08.048 PMC7115487

[10] Klink T , Rankin DA , Piya B , et al. Evaluating the diagnostic accuracy of the WHO Severe Acute Respiratory Infection (SARI) criteria in Middle Eastern children under two years over three respiratory seasons. PLoS One. 2020;15(4):e0232188. 32353012 10.1371/journal.pone.0232188 PMC7192447

[11] Miller EK , Khuri‐Bulos N , Williams JV , et al. Human rhinovirus C associated with wheezing in hospitalised children in the Middle East. J Clin Virol. 2009;46(1):85‐89. 19581125 10.1016/j.jcv.2009.06.007 PMC2759319

[12] Harris PA , Taylor R , Thielke R , Payne J , Gonzalez N , Conde JG . Research electronic data capture (REDCap)–a metadata‐driven methodology and workflow process for providing translational research informatics support. J Biomed Inform. 2009;42(2):377‐381. 18929686 10.1016/j.jbi.2008.08.010 PMC2700030

[13] Al Amad MA , Al Mahaqri AA , Al Serouri AA , Khader YS . Severe acute respiratory infections with influenza and Noninfluenza respiratory viruses: Yemen, 2011–2016. Inquiry. 2019;56:46958019850731. 31137990 10.1177/0046958019850731 PMC6542124

[14] Caini S , El‐Guerche Séblain C , Ciblak MA , Paget J . Epidemiology of seasonal influenza in the Middle East and North Africa regions, 2010–2016: circulating influenza A and B viruses and spatial timing of epidemics. Influenza Other Respir Viruses. 2018;12(3):344‐352. 29405575 10.1111/irv.12544 PMC5907816

[15] Refaey S , Hassan M , Mansour A , Kandeel A . Incidence of influenza virus‐associated severe acute respiratory infection in Damanhour district, Egypt, 2013. East Mediterr Health J. 2016;22(7):503‐512. 27714745

[16] Zhang T , Zhu Q , Zhang X , et al. The clinical characteristics and direct medical cost of influenza in hospitalized children: a five‐year retrospective study in Suzhou, China. PLoS One. 2012;7(9):e44391. 22957069 10.1371/journal.pone.0044391 PMC3434134

[17] Gouya M , Rezaei F , Haghdoost A , et al. Estimation of influenza and severe acute respiratory illness incidence (burden) in three provinces of the Islamic Republic of Iran, 2012 and 2013. East Mediterr Health J. 2016;22(7):432‐439. 27714736 10.26719/2016.22.7.432

[18] Kandeel A , Dawson P , Labib M , et al. Morbidity, mortality, and seasonality of influenza hospitalizations in Egypt, November 2007‐November 2014. PLoS One. 2016;11(9):e0161301. 27607330 10.1371/journal.pone.0161301 PMC5015910

[19] Refaey S , Amin M , Labib M , Kandeel A . Influenza virus positivity and circulating subtypes among cases of influenza‐like illness and severe acute respiratory infection, Egypt, 2012–2015. East Mediterr Health J. 2016;22(7):527‐536. 27714747

[20] Silvennoinen H , Peltola V , Vainionpää R , Ruuskanen O , Heikkinen T . Admission diagnoses of children 0–16 years of age hospitalized with influenza. Eur J Clin Microbiol Infect Dis. 2012;31(3):225‐231. 21643867 10.1007/s10096-011-1297-8

